# Incisional Hernia Involving the Neobladder: Technical Considerations to Avoid Complications

**DOI:** 10.1100/tsw.2009.84

**Published:** 2009-06-30

**Authors:** Devendar Katkoori, Anuradha Jayathillake, Ahmed Eldefrawy, Murugesan Manoharan

**Affiliations:** Department of Urology, Miller School of Medicine, University of Miami, FL, USA

**Keywords:** dual-layer mesh, incisional hernia, mesh erosion, neobladder, omental flap, urinary diversion

## Abstract

The management of incisional hernia following radical cystectomy (RC) and neobladder diversion poses a special challenge. Mesh erosion into the neobladder is a potential complication of hernia repair in this setting. We describe our experience and steps to avoid this complication. Three patients developed incisional hernias following RC involving the neobladder. The incisional hernias were repaired by the same surgeon. A systematic dissection and repair of the hernias with an onlay dual-layer mesh (made of polyglactin and polypropylene) was carried out. The critical steps were placing the polyglactin side of the mesh deeper and positioning of an omental flap anterior to the neobladder. The omental flap adds a protective layer that prevents adhesions between the neobladder and abdominal wall, and prevents erosion of the mesh into the fragile neobladder wall. All of these patients had received two cycles of neoadjuvant chemotherapy prior to RC. The time duration from RC to the repair of hernia was 7, 42, and 54 months. No intraoperative injury to the neobladder or other complication was noted during hernia repair. The patients were followed after hernia repair for 20, 22, and 42 months with no recurrence, mesh erosion, or other complications. Careful understanding and attention to details of the technique can minimize the risk of complications, especially incisional hernia recurrence, injury to the neobladder, and erosion of mesh into the neobladder wall.

## INTRODUCTION

Radical cystectomy (RC) is a frequently performed surgery for muscle-invasive bladder cancer. Urinary diversion is concurrently performed. Neobladder urinary diversion has become a widely accepted form of diversion and is presently the most common form of diversion[1,2,3]. With this trend, clinicians have become increasingly familiar with surgical and medical complications related to this form of urinary diversion[4]. Incisional hernia is a well-described surgical complication of abdominal surgery, with an incidence of 2–11%[5]. Incisional hernia has a multifactorial etiology. Incisional hernia involving the reconstructed bladder poses a unique challenge to the urologist. Re-entry into the abdomen is difficult; the wall of the sac may be adherent to the neobladder with potential for injury to the neobladder. Also, mesh repair has potential for complications in that the mesh can erode into the thin neobladder wall. We describe our experience with incisional hernia involving the neobladder, and discuss technical considerations and prevention of complications.

## METHODS

A total of three patients (one female, two males) had RC at our institution and later presented with incisional hernia involving the neobladder. A complete preoperative assessment was performed. Cross-sectional imaging was used to determine the extent of the neobladder involvement in the hernia sac. We used the technique outlined below for incisional hernia repair in these patients.

The patient was placed in the supine position. Broad spectrum antibiotic cover was used. A Foley catheter was introduced to decompress the neobladder. The incision was made, excising the existing scar, and progressed to identify the fascial defect in the anterior rectus sheath. The edge of the hernia was defined by undermining the subcutaneous tissue at this level. Two patients had the repair performed extraperitoneally; in the third patient, the peritoneal cavity was opened. The dissection then progressed caudal to separate the neobladder from previously placed omentum.

A dual-layer mesh was fashioned by bringing together a Vicryl® woven mesh (polyglactin910, Ethicon) and a Prolene® mesh (polypropylene, Ethicon), using 2/0 Prolene® sutures. The composite mesh was then placed with the Vicryl® side of the mesh deeper, in onlay fashion, and sutured using 2/0 Prolene®. Imbricating sutures incorporated into the hernia repair were used to give more strength. Copious neomycin antibiotic irrigation was used to wash the wound thoroughly. A systematic layered closure was carried out and no drain was placed.

## RESULTS

Three patients underwent hernia repair involving the neobladder for moderate-size incisional hernia over a period of 10 years from 1997 to 2007. They previously received two cycles of neoadjuvant chemotherapy with gemcitabine and cisplatin, and had RC with neobladder urinary diversion for urothelial carcinoma of the bladder at our institution. There were no specific complications at the time of the cystectomy to predispose these patients to incisional hernias. All the patients had an uneventful recovery following RC. Two patients had a history of coronary vascular disease. Of note is the fact that all three patients were exsmokers. The time duration from RC to the repair of hernia was 7, 42, and 54 months.

All patients had an uneventful incisional hernia repair, with no intraoperative injury to the neobladder or other complications. The patients were followed after hernia repair for 20, 22, and 42 months (mean: 28 months) with no recurrence, mesh erosion, or other complications.

## DISCUSSION

The use of mesh for hernia repair was first reported by Usher et al.[6]. Using mesh for hernia repair revolutionized the success rate and reduced the recurrence rate from 50% to less than 10%[5,7,8]. Multiple techniques have been utilized for incisional hernia repair including primary closure, onlay/inlay mesh reinforcement, retrorectus mesh placement, and intraperitoneal mesh placement, or combinations of the above repairs[5].

An incisional hernia involving the neobladder is a unique situation and deserves special precautions. Several steps facilitate the hernia repair and avoid complications. Placing a catheter ensures that the neobladder is decompressed during the repair and should be retained until the patient ambulates. If entry onto the peritoneal cavity is required for the repair, it has to be entered well above the neobladder at the upper part of the incision. Exposing the symphysis pubis is important, as this will allow placement of sutures later in the procedure to hold the mesh. However, the neobladder can drape over this structure within the hernia and adequate care should be taken to avoid inadvertent injury.

The onlay technique is preferred, as it keeps the mesh separated from abdominal contents and avoids erosion of the mesh into the viscera[7]. The sublay technique is not recommended due to lack of arcuate fascia below the umbilicus, which places the mesh in close approximation to the viscera, increasing the risk for migration of the mesh and erosion into the neobladder[7]. Erosion of the mesh into the native urinary bladder has been reported previously[7]. We believe that the risk of erosion of the mesh into the fragile reconstructed bladder is relatively higher than for the native bladder. Hence, we use a dual-layer mesh with an inner polyglactin layer and also place an omental flap anterior to the neobladder to prevent mesh erosion. The mesh should never be cut because this will create sharp edges that can cause an inflammatory reaction, predisposing to erosion into the neobladder or other viscera[7]. Proper placement of sutures is essential to avoid migration of the mesh.

The risk of incisional hernia after RC can be reduced by taking adequate intraoperative precautions. We take extra care to ensure systematic mass closure of the abdominal wall. Fascial edges are carefully identified. A viscus-retaining device (Fish Glassman retainer, Adept Med International, Diamond Springs, CA) is routinely used in all abdominal closures. Placing an omental flap anterior to the neobladder provides another protective layer to the fragile reconstructed bladder.

In patients with sizable omentum, we initially mobilize the omentum on the left gastroepiploic pedicle and then divide the omental wrap into two segments, placing one leaf anterior and another posterior to the neobladder. Placing a single retaining suture posterior to the urethra facilitates the posterior leaf placement. In the unusual event of an incisional hernia developing at a later period, this further layer protects the neobladder from injuries during dissection and repair. Adhesions between the omental flap and the abdominal wall can be dissected with no significant risk of injuring the neobladder.

## CONCLUSIONS

Although incisional hernia involving the neobladder following RC is a rare complication, we believe this complication will become more prevalent as neobladder urinary diversion becomes widely used. Careful attention and understanding of the technique described will lead to successful repair by minimizing the chance of recurrence, erosion of the mesh into the neobladder wall, or other complications.

## ACKNOWLEDGMENTS

Support was received from "CURED" and Mr. Vincent A. Rodriguez.
